# Dynamic cyber risk estimation with competitive quantile autoregression

**DOI:** 10.1007/s10618-021-00814-z

**Published:** 2022-01-04

**Authors:** Raisa Dzhamtyrova, Carsten Maple

**Affiliations:** 1 The Alan Turing Institute, London, United Kingdom; 2 Department of Computer Science, Royal Holloway, University of London, London, United Kingdom; 3 WMG Cyber Security Centre, University of Warwick, London, United Kingdom

**Keywords:** Cyber risk, Dynamic risk estimation, Time-series, Quantile Autoregression, Competitive prediction, Cyber breach modelling

## Abstract

The increasing value of data held in enterprises makes it an attractive target to attackers. The increasing likelihood and impact of a cyber attack have highlighted the importance of effective cyber risk estimation. We propose two methods for modelling Value-at-Risk (VaR) which can be used for any time-series data. The first approach is based on Quantile Autoregression (QAR), which can estimate VaR for different quantiles, i. e. confidence levels. The second method, we term Competitive Quantile Autoregression (CQAR), dynamically re-estimates cyber risk as soon as new data becomes available. This method provides a theoretical guarantee that it asymptotically performs as well as any QAR at any time point in the future. We show that these methods can predict the size and inter-arrival time of cyber hacking breaches by running coverage tests. The proposed approaches allow to model a separate stochastic process for each significance level and therefore provide more flexibility compared to previously proposed techniques. We provide a fully reproducible code used for conducting the experiments.

## Introduction

The prevalence and impact of cyber attacks on organisations are increasing at an alarming rate. Risk estimation is an important task for any company or institution, allowing them to predict and assess adverse events which can lead to financial and reputation losses, enabling them to plan for and mitigate against these threats through effective risk management.

Kaplan and Garrick (1981) define risk to be a set of triplets, which consist of a risk scenario description, the probability of that scenario, and the consequence or evaluation measure of that scenario, i.e., a measure of damage. Another definition of risk is provided by Holton (2004), in which the risk comprises two components: uncertainty and exposure. Indeed all definitions of risk require some form of assessment of the likelihood of adverse events and their severity. A recent report by Jones and Tivnan (2018) from the Department of Homeland Security provides a survey of risk metric frameworks and risk models. One of the quantitative risk metrics described in the report is Cyber Value-at-Risk (VaR), an adaptation of the financial VaR, for the quantification of cyber security risk. VaR is one of the most important risk measurements in finance and involves measuring the maximum loss over a preset horizon with a pre-defined confidence level (Hull 2006). VaR has now found various applications in cyber security areas. For example, Factor Analysis of Information Risk (FAIR), considered “an international standard information risk management model”, is based on VaR. FAIR is defined as “a standard Value-at-Risk model for information and operational risk that helps information risk, cyber security and business executives measure, manage, and communicate on information risk in a language that the business understands, dollars and cents” (Jones and Tivnan 2018). Peng et al. (2016) used VaR to estimate the probability of extreme cyber attacks over a pre-defined period of time. Raugas et al. (2013) proposed a model to quantify the monetary VaR due to cyber threats based on the Bayesian networks. The detailed model described the example attack graph of unauthorized access to intellectual property. In this paper, we propose a new methodology of estimation of VaR for cyber events.

In this paper, we aim to provide a framework that can model risks *dynamically* and re-estimate cyber risk when new data becomes available. Many current risk methods are based on manual risk analysis during the system’s design process. Some of the examples of traditional qualitative methods include scenario analysis and questionnaires, which are heavily dependent on experts’ subjective opinions. On the other hand, quantitative risk methods are usually based on unreliable data, and therefore their precision is prone to errors (Taubenberger et al. 2011). As a result, there is a lack of current research on dynamic cyber risk estimation. Of the work that has been proposed for dynamic risk modelling, a number of approaches are based on Hidden Markov Models (HMM). Arnes et al. (2005) proposed a real-time risk estimation method, which aggregates data from several intrusion detection systems allowing dynamic estimation of systemic risk using HMM. Li et al. (2018) developed a method to dynamically model the risks of users’ activity patterns in social networks. The approach is based on HMM and Bayesian Risk Graph model. Unlike the previous approaches, we do not model the dynamics of the system states with HMM. Instead, we focus on time-series data and propose a new method for dynamic estimation of VaR. System monitoring is essential to effective risk governance. The monitored data is usually a different kind of time-series, such as various sensor data, login data, and intrusion and hacking attempts. From a risk perspective, it is critical to estimate the probability of extreme events. For example, we do not want to predict the mean or the median of hacking attempts over a pre-defined period. Instead, we aim to assess the maximum number of hacking attempts with the desired confidence. For this purpose, we suggest to model VaR as a quantile of time-series, where each quantile corresponds to the desired confidence level. These values of VaR can also be translated into the monetary equivalent. For example, if we assume that each cyber hacking attempt costs one pound for a company, we can estimate the budget allocation which should be devoted to security defence. The proposed method builds upon the Weak Aggregating Algorithm for Quantile Regression (WAAQR) (Dzhamtyrova and Kalnishkan 2020) and is adapted to the case of time-series forecasting.

## Related work

In this paper, we propose a new framework for dynamic estimation of VaR. Though the proposed methods can be used to predict any types of time-series, we perform our experiments on the Privacy Rights Clearinghouse (PRC), which contains the chronology of the reported data breaches since January 2005.^1^ The reasons are that this dataset contains one of the largest cyber events data available online, it is regularly updated, and it was studied before in the literature. Our analysis closely resembles the analysis of Xu et al. (2018), however, we propose different modelling approaches of hacking breaches. First, our methods can be applied to any kind of time-series data. Second, the analysis of Xu et al. (2018) models the mean of inter-arrival times and sizes, and then VaR is found by simulating 10,000 samples based on the estimated copula. Instead, we suggest that each quantile of inter-arrival times and sizes of cyber incidents can be modelled with separate stochastic processes. Though we do not investigate the relationship between inter-arrival times and sizes of breaches, we argue that the proposed methods are more flexible in comparison to previous research as they make fewer assumptions on the nature of the data, since each quantile of breach size or inter-arrival time can be modelled with a separate stochastic process. In our experiments, we first show that we can apply Quantile Autoregression (QAR) (Koenker and Xiao 2006) to estimate VaR of hacking breaches. The Basel Committee recommends assessing the quality of the VaR models by running some form of backtesting. Standard backtesting methods include the Kupiec unconditional coverage test (Kupiec 1995) and the Christoffersen conditional coverage test (Christoffersen 1998). We apply both tests to assess the performance of QAR. The results show that for breach size QAR fits well, and for an inter-arrival time, it rejects the null hypothesis of the conditional coverage test of violation occurrence for one considered quantile. We then propose a new framework, Competitive Quantile Autoregression (CQAR), which improves the prediction of hacking breach inter-arrival times.

The proposed method CQAR is based on the competitive prediction approach, where one algorithm ‘competes’ with other predictive algorithms. The goal is to provide a strategy that can guarantee a performance close to the best predictive models. To solve the problem of competitive prediction, the Aggregating Algorithm (AA) was proposed by Vovk (1990). The AA mixes the predictions of a number of models in a similar manner to the Bayesian method, where the prediction is calculated based on the model’s prior distribution and the data likelihood. Furthermore, the AA guarantees that the loss of the resulting mixing strategy is as small as the best model’s plus a constant for any time point in the future. The Weak Aggregating Algorithm (WAA) was proposed by Kalnishkan and Vyugin (2008) as an alternative for the AA, which provides better theoretical guarantees for some loss functions, such as the pinball loss, which we consider in this paper. In the general case, both the AA and the WAA mix and compete with a finite number of algorithms.

It is possible to construct strategies that combine infinite classes of functions and provide theoretical guarantees compared to these classes. The Aggregating Algorithm for Regression chooses the competitor strategies to be all linear functions (Vovk 2001). The resulting strategy asymptotically performs as well as any linear regression in terms of the cumulative square loss. A similar approach is undertaken by Dzhamtyrova and Kalnishkan (2020) to propose the Weak Aggregating Algorithm for Quantile Regression. The strategy is a Bayesian mixture, which combines an infinite pool of quantile regressions, and asymptotically predicts as well as any of them in terms of the cumulative pinball loss. The algorithm was previously applied to probabilistic forecasting of renewable energy where the prediction of renewable energy was made based on the weather data; the approach showed a good performance. The proposed algorithm CQAR is built on the WAAQR algorithm and is adapted to time-series forecasting. Instead of mixing a class of quantile regressions, we suggest combining a class of QAR. It also has the property that it asymptotically predicts as well as any QAR. We provide the pseudo-code of CQAR, which uses Metropolis-Hastlings sampling (Andrieu et al. 2003) to calculate its predictions, however, it can be substituted with any other sampling algorithm. We show that CQAR produces better results in comparison to QAR for estimating VaR of hacking breach inter-arrival times. Another advantage of CQAR is that it re-estimates cyber risks dynamically after new observations become available. We also plot the average regret between CQAR and the best QAR depending on time and show that it goes to zero as time increases. This empirically confirms the theoretical guarantees of the method and shows that CQAR is asymptotically as good as the best QAR which was trained on the training dataset.

## Contributions

Our first contribution is a new analysis and adaptation of QAR for calculating cyber VaR. To the best of our knowledge, it was not done before. The method can be applied to any time-series data. It is common to predict the mean or median values of time-series. Some research also focuses on the prediction of extreme values. This analysis provides a new way to model extreme values that also comes with the desired confidence level. QAR allows to model VaR for each confidence level with a separate stochastic process, and hence allows more flexibility compared to previously proposed approaches in the literature.

The second contribution is a new dynamic risk estimation method, Competitive Quantile Autoregression (CQAR). There is a lack of research on dynamic cyber risk estimation. CQAR allows to re-estimate cyber risk at each time step when new data becomes available and works for any time-series data. An important property of this approach is its theoretical guarantee that it asymptotically predicts as well as the best QAR. The theoretical performance guarantees provide confidence in the prediction as they will hold for any new unseen data, while at the same time the method allows adapting to a changing environment. As with QAR, CQAR is also more flexible as it models each quantile with a separate stochastic process.

The third contribution is the modelling of cyber data breaches with the proposed methods. We show that both QAR and CQAR can be used to estimate VaR of cyber breaches’ sizes and inter-arrival times. The coverage tests show a good fit of both approaches. We show that CQAR provides better results for modelling hacking breaches’ inter-arrival times compared to QAR. We also illustrate the behaviour of the average regret between CQAR and QAR during the time and show that it conforms to the theoretical bounds of CQAR. The comparison of CQAR and ARMA(1, 1)-GARCH(1, 1) shows that the methods are on par with each other even though CQAR uses much smaller data for training. The fully reproducible open-source code of our implementation is available at GitHub.^2^

## Risk estimation with Quantile Autoregression

VaR is a widely used risk measurement in finance. ${{\,\mathrm{VaR}\,}}_{\alpha }$ is defined as the loss corresponding to the $\alpha$-quantile of the distribution of the gain in the value of the portfolio over the next *N* days (Chapter 21.1 in Hull (2006)). In finance, VaR provides an estimate of the maximum loss for a certain confidence level and is important for budget allocation and financial reserves. Analogously, in cyber security, we want to estimate possible losses of extreme cyber events, such as cyber attacks and subsequent data losses. Accurate forecasting of these adverse events can allow an adaptation of risk mitigation strategies and better financial planning.

Let the outcomes have a cumulative distribution $F_Y(z)$, then we define

1 $$\begin{aligned} {{\,\mathrm{VaR}\,}}_{\alpha } = \inf \{z: F_Y(z) \ge \alpha \} \end{aligned}$$

as the $\alpha$-quantile of *Y*. Then we can estimate ${{\,\mathrm{VaR}\,}}_\alpha$ as $\alpha$-quantile of outcomes.

QAR, proposed by Koenker and Xiao (2006), allows to model each quantile of outcomes with a separate autoregressive process. Let time-series $y_t$ to be the *p*-order autoregressive process:

2 $$\begin{aligned} y_t = \theta _0(U_t) + \theta _1(U_t) y_{t-1} + \cdots + \theta _p(U_t) y_{t-p}, \end{aligned}$$

where $\{U_t\}$ is a sequence of i.i.d. standard uniform random variables. We want to estimate the coefficients $\theta _j$, which are unknown functions $[0, 1] \rightarrow \mathbb {R}$. The $\alpha$th conditional quantile of $y_t$ is:

3 $$\begin{aligned} Q_{y_t}(\alpha | y_{t-1}, y_{t-2}, \ldots , y_{t-p}) = \theta _0(\alpha ) + \theta _1(\alpha ) y_{t-1} + \cdots + \theta _p(\alpha ) y_{t-p}. \end{aligned}$$

Equation (3) can be rewritten in analogous to the definition of quantile regression (Koenker and Bassett 1978):

4 $$\begin{aligned} Q_{y_t}(\alpha | \mathcal {F}_{t-1}) = x_t^\prime \theta (\alpha ), \end{aligned}$$

where $x_t = (1, y_{t-1}, \ldots , y_{t-p})^\prime$, $\theta = (\theta _0, \theta _1, \ldots , \theta _{t-p})^\prime$, and $\mathcal {F}_{t-1}$ is the $\sigma$-field generated by $\{y_s, s \le t\}$.

The coefficients $\theta (\alpha )$ in (4) are found by minimising the following expression:

5 $$\begin{aligned} \min _{\theta \in \mathbb {R}^{p+1}} \sum _{t} \lambda (y_t, x_t^\prime \theta ), \end{aligned}$$

where $\lambda (y, \gamma )$ is the pinball loss function:

6 $$\begin{aligned} \lambda (y, \gamma ) = {\left\{ \begin{array}{ll} \alpha (y - \gamma ), &{} \text {if } y \ge \gamma \\ (1-\alpha ) (\gamma - y), &{} \text {if } y < \gamma \end{array}\right. }. \end{aligned}$$

## Framework of competitive prediction

In this section, we describe the framework of competitive prediction. In this framework, a *learner* plays a *game* $\mathfrak {G}$ against other prediction strategies and a *nature*, which reveals the true outcomes. A game $\mathfrak {G} = \langle \varOmega , \varGamma , \lambda \rangle$ is a tuple with the space of outcomes $\varOmega$, decision space $\varGamma$, and a loss function $\lambda$. In this paper, we consider $\varOmega = \varGamma = \mathbb {R}$, and $\lambda$ to be the pinball loss, defined in (6) for $\alpha \in (0, 1)$.

The learner works according to the following protocol:

### Protocol 1


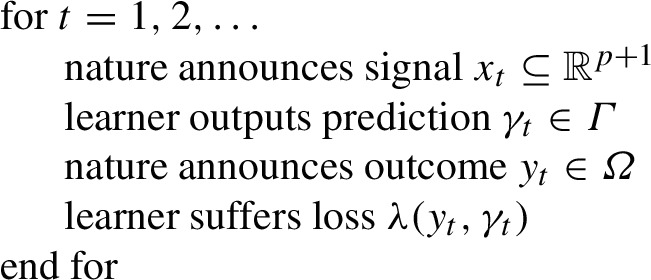


Before seeing the true outcome $y_t \in \varOmega$, the learner needs to make a prediction $\gamma _t \in \varGamma$, based on a signal $x_t$, which is announced by nature. After seeing the true outcome $y_t$, the learner’s loss $\lambda (y_t, \gamma _t)$ can be calculated.

In this paper, we assume that the outcomes follow the *p*-order autoregressive process defined in (2). The learner makes a prediction $\gamma _t$ based on the signal $x_t = (1, y_{t-1}, \ldots , y_{t-p}) \in \mathbb {R}^{p+1}$. For ease of notation, we replace $\theta (\alpha )$ with $\theta$. Let us denote $\xi _t(\theta )$ to be the prediction (4) of QAR(p):

7 $$\begin{aligned} \xi _t(\theta ) = x_t^\prime \theta . \end{aligned}$$

We denote the cumulative loss of the learner at step *T* as:

$$\begin{aligned} L_T := \sum _{t=1}^T \lambda (y_t, \gamma _t) = \sum _{\begin{array}{c} t = 1, \ldots , T:\\ {y_t > \gamma _t} \end{array}} \alpha |y_t - \gamma _t| + \sum _{\begin{array}{c} t = 1, \ldots , T:\\ y_t < \gamma _t \end{array}} (1-\alpha ) |y_t - \gamma _t|. \end{aligned}$$

The cumulative loss of the prediction strategy $\mathcal {E}_\theta$, parametrised by $\theta$, which at step *T* outputs $\xi _t(\theta )$:

8 $$\begin{aligned} L_T^{\theta }:= & {} \sum _{t=1}^T \lambda (y_t, \xi _t(\theta )) = \sum _{\begin{array}{c} t = 1, \ldots , T:\\ {y_t > \xi _t(\theta )} \end{array}} \alpha |y_t - \xi _t(\theta )| \nonumber \\&+\sum _{\begin{array}{c} t = 1, \ldots , T:\\ y_t < \xi _t(\theta ) \end{array}} (1-\alpha ) |y_t - \xi _t(\theta )| . \end{aligned}$$

Our goal is to find a strategy which at time *t* can compete with any prediction strategy $\xi _t(\theta )$ in terms of cumulative losses.

We denote the *regret* at time *T* to be the difference between the cumulative losses of the learner and the prediction strategy $\mathcal {E}_\theta$:

9 $$\begin{aligned} R_T = L_T - L_T^{\theta }, \end{aligned}$$

and the *average regret* at time *T* to be:

10 $$\begin{aligned} \hat{R}_T = \left( L_T - L_T^{\theta } \right) / T. \end{aligned}$$

## Competitive Quantile Autoregression

In this section, we describe CQAR, which is an adaptation of WAAQR (Dzhamtyrova and Kalnishkan 2020) to time-series forecasting. The algorithm works according to Protocol 1, which is different from the traditional machine learning approach, where one needs a dataset for the algorithm’s training. CQAR makes its prediction based on the signal, which is announces by the nature. We assume that the outcomes follow *p*-order autoregressive process (2). At the time step *T* we observe signal $x_T = (1, y_{T-1}, \ldots , y_{T-p})$, which contains *p* previous outcomes. Based on this signal, we need to output the prediction $\gamma _T$ before seeing the true outcome $y_T$. In contrast to QAR, CQAR does not try to find the optimal parameters $\theta$ by minimising the pinball loss function (5). Instead, CQAR combines the predictions of a large pool of QAR in a way, which is similar to a Bayesian mixture.

We show how CQAR is derived from Weak Aggregating Algorithm (WAA) (Kalnishkan and Vyugin 2008) which updates the weights of strategy $\mathcal {E}_\theta$ at step *t* according to its loss:

11 $$\begin{aligned} P_t(d\theta ) = \exp \left( \frac{-c L_{t-1}^{\theta }}{\sqrt{t}} \right) P_0(d\theta ), \end{aligned}$$

where $P_0(d\theta )$ is the initial weight of prediction strategy $\mathcal {E}_\theta$ and *c* is a positive constant. The prediction of WAA at step *t* is a weighted average of strategies predictions $\xi _t(\theta )$:

12 $$\begin{aligned} \gamma _t = \int _{\varTheta } \xi _t(\theta ) P_{t-1}^*(d\theta ), \end{aligned}$$

where $\varTheta$ is a parameter space, i.e. $\theta \in \varTheta$ and $P_{t-1}^*(d\theta )$ are normalised weights of strategy $\mathcal {E}_\theta$ at step $t-1$:

13 $$\begin{aligned} P_{t-1}^*(d\theta ) = \frac{P_{t-1}(d\theta )}{P_{t-1}(\varTheta )}. \end{aligned}$$

We choose an initial distribution of parameters

14 $$\begin{aligned} P_0(d\theta ) = \left( \frac{a}{2} \right) ^{p+1} e^{-a \Vert \theta \Vert _1}d\theta , \end{aligned}$$

for some $a>0$, and $\theta \in \varTheta = \mathbb {R}^{p+1}$. Then by putting (14) and (11) in (13) the normalised weights of strategy $\mathcal {E}_\theta$ at step *t*:

15 $$\begin{aligned} P_t^*(d\theta ) = Z \exp \left( -\frac{L_{t-1}^{\theta }}{\sqrt{t}} - \frac{a}{c} \Vert \theta \Vert _1\right) = Z \exp \left( -\frac{L_{t-1}^{\theta }}{\sqrt{t}} - \hat{a} \Vert \theta \Vert _1\right) , \end{aligned}$$

where *Z* is the normalising constant ensuring that $\int _{\mathbb {R}^{p+1}} P_t^{*}(d\theta ) = 1$. By putting this expression in (12) and putting the cumulative loss of the strategy $\mathcal {E}_\theta$ from (8) we get the prediction of CQAR at step *T*:

16 $$\begin{aligned} \gamma _T = \int _{\varTheta } \xi _T(\theta ) q_{T-1}^{*}(\theta ) d\theta , \end{aligned}$$

where

17 $$\begin{aligned} q_T^*(\theta )= & {} Z q_T (\theta )= Z \exp \Bigl ( -\frac{1}{\sqrt{T}} \Bigl (\sum _{\begin{array}{c} t = 1, \ldots , T:\\ y_t < \xi _t(\theta ) \end{array}}(1-\alpha ) |y_t - \xi _t(\theta )| \nonumber \\&+ \sum _{\begin{array}{c} t = 1, \ldots , T:\\ y_t > \xi _t(\theta ) \end{array}} \alpha |y_t - \xi _t(\theta )|\Bigr ) -a\Vert \theta \Vert _1 \Bigr ), \end{aligned}$$

where *a* is a regularisation parameter and *Z* is the normalising constant ensuring that $\int _\varTheta q_T^{*}(\theta )d\theta = 1$, and $\Vert \theta \Vert _1$ denotes $L_1$-norm of parameter $\theta$. Function $q_T^*(\theta )$ has a meaning of the likelihood of the parameters $\theta$ at time step *T*. The pseudo-code of CQAR uses the Metropolis-Hastings algorithm, which is a Markov chain Monte Carlo (MCMC) method (Andrieu et al. 2003), though any other sampling algorithm could be used instead to approximate the integral (16). We start with some initial parameter $\theta ^0$ and at each step *m* we update:

$$\begin{aligned} \theta ^m = \theta ^{m-1} + \mathcal {N} (0, \sigma ^2),~ m=1,\ldots , M, \end{aligned}$$

where $\mathcal {N}(0, \sigma ^2)$ is the Gaussian proposal distribution with standard deviation $\sigma$, and *M* is the total number of MCMC iterations. The Metropolis-Hastings randomly walks through the parameter space $\varTheta$, and either accepts or rejects new parameters $\theta$. If the likelihood of the new parameters (17) is higher than the old parameters’ likelihood, the new parameters are always accepted. Otherwise, the new parameters can be either accepted or rejected. By moving this way, the algorithm mostly samples parameters $\theta$ from the high-density regions of (17), only sometimes visiting the area of low-density of the parameters’ likelihood. This procedure allows giving an accurate approximation of the integral (16).

We provide the pseudo-code of CQAR below. The algorithm has four input parameters: the number of MCMC iterations *M*, the ‘burn-in period’ $M_0$, the regularisation parameter *a*, and the standard deviation $\sigma$. The burn-in period $M_0$ means that we sample $M_0$ values of the parameters, but they are not used in the integral approximation. It is useful as we probably did not yet reach the area of high density of the parameters’ likelihood. 
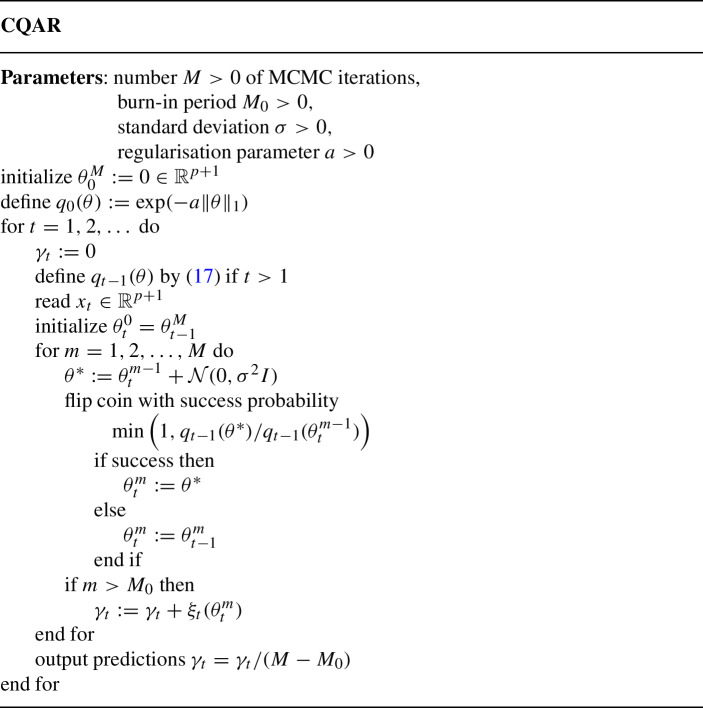


An important property of CQAR is that it asymptotically predicts as well as the best QAR. The following theorem provides the upper bound for the average regret between CQAR and the best QAR.

### Lemma 1

(Theorem 1 in Dzhamtyrova and Kalnishkan (2020)) Let $a > 0$, $A \le y_t \le B$ for any $t=1, 2, \ldots , T-1$, where *T* is a positive integer. For every sequence of outcomes of length *T*, and every $\theta \in \mathbb {R}^{p+1}$ the average regret $\hat{R}_T$ between CQAR and QAR satisfies

$$\begin{aligned} \hat{R}_T \le \frac{1}{\sqrt{T}} a \Vert \theta \Vert _1 + \frac{1}{\sqrt{T}} \Bigg ((p+1) \ln \left( 1 + \frac{\sqrt{T}}{a} \max (1, B) \right) + (B-A)^2 \Bigg ). \end{aligned}$$

The derivation of Lemma 1 can be found in the Appendix. The theorem states that CQAR asymptotically predicts as well as the best QAR as the average regret $\hat{R}_T \rightarrow 0$, for $T \rightarrow +\infty$. Although the bound contains the information about the minimum and maximum values of the outcomes at the previous steps, it does not affect the asymptotic behaviour of the bound. The choice of the regularisation parameter *a* affects the behaviour of the theoretical bound. As a result, it is important to pick the parameter which minimizes the regret’s bound. However, in most cases, the optimal choice of the regularisation parameter cannot be found in advance as the number of steps *T* is usually not known from the start. We discuss the choice of the parameters of CQAR in detail in the experimental part of the article.

## Experiments

We apply the proposed approach to the prediction of cyber hacking breaches. The data is taken from the PRC report $^{1}$, which contains the chronology of the reported data breaches since January 2005. This benchmark dataset has been used by a number of other researchers in establishing the efficacy of their work (Edwards et al. 2016; Xu et al. 2018). The analysis of data breaches is attracting research activity lately, given the importance of the topic. Some of this work suggests that data breaches can be modelled using a variety of distributions. Hubbard and Seiersen (2016) suggest using the beta distribution for estimating the probability of data breaches based on industry data. After estimating the probability of data breaches, the VAR is modelled with the Monte-Carlo simulation, which gives a forecast of the possible losses. Edwards et al. (2016) investigate the PRC dataset from the period between January 2005 and September 2015. The study examined over 20 different distributions, such as log-normal, power-law, generalised Pareto to determine which provided the best fit for the size of the data breach. To model the breach frequencies, the authors investigated a number of discrete distributions, such as Poisson, binomial, and negative binomial. The results suggest that neither the size nor the frequency of data breaches has increased over the period under consideration. Furthermore, the study proposes to model the daily frequency of breaches using the negative binomial distribution, whereas breach sizes are best described by the log-normal family of distributions. It is, of course, possible that the nature of data breaches has changed significantly in the era of increasing data connectivity. Xu et al. (2018) analyse the PRC dataset with a focus on hacking breach incidents. Their analysis shows that both the inter-arrival time and the size of hacking breaches reveal significant auto-correlation and partial auto-correlation, suggesting that the breaches can be modelled with stochastic processes. The paper estimates the inter-arrival times with the autoregressive conditional mean model, whereas the breach sizes are estimated with ARMA(1, 1)-GARCH(1, 1). The authors also show that there is a positive correlation between inter-arrival times and sizes of cyber incidents, and describe this dependence by a particular copula.

We use the same benchmark to evaluate the performance of the proposed approaches. The open-source code of our implementation is fully reproducible and available at GitHub $^{2}$. The data contains the chronology of various types of data breaches such as card fraud, insider incidents, paper, and computer physical losses, and unintended information disclosure. The companies which suffer the incidents are classified into seven types of businesses: BSF (Financial and Insurance Services Businesses), BSR (Retail/Merchant including Online Retail Businesses), BSO (Other Businesses), EDU (Educational Institutions), GOV (Government and Military), MED (Medical and Healthcare), and NGO (Nonprofits). The report contains 9015 data breaches between January 2005 and September 2019. The top three types of reported breaches are: data hacked or infected by malware (HACK −28.1%), unintended disclosure of sensitive information posted publicly, mishandled or sent to the wrong party (DISC −20.6%), information lost or stolen from paper documents (PHYS −19.2%).

### Hacking breaches

In this section, analogous to Xu et al. (2018), we focus on the largest type of reported data breaches: hacking breaches. The total number of observations after removing all incomplete, unknown, and missing breaches is 1602. We hold the first 60% of the data for training and the last 40% for testing: the size of the training set is 956, whereas that of the test set is 636.

#### Data exploration

We start with data pre-processing. Most days have only one incident per day, 232 days have two incidents, 52 days have three, and 35 days are with more than three incidents. Similarly to Xu et al. (2018), if several events occur in one day, they are analysed as separate incidents. For these events, we generate a random number from zero to one, which corresponds to some time during the day. After that, these events are sorted by these randomly generated numbers.

**Fig. 1:**
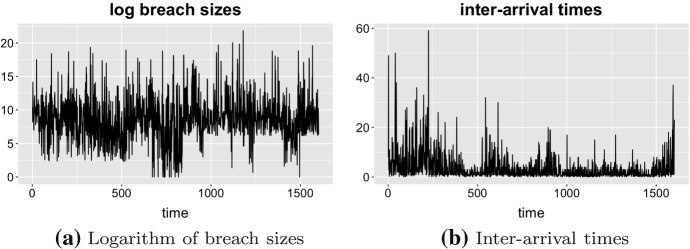
Visualisation of breach sizes and inter-arrival times

Figure 1 visualises inter-arrival times and the logarithm of breach size, where size is the total number of accounts affected by the breach. We visualise breach sizes on a logarithmic scale because some of the incidents exhibit particularly extreme values. Table 1 describes the summary statistics of breach sizes, where sd denotes the standard deviation. The analysis in Xu et al. (2018) describes the period between January 2005 and April 2017 and contains 600 hacking breaches. We observe that more than 1000 incidents have been added to the report in the last two years. It indicates that either hacking incidents become more frequent or the companies become more transparent about reporting their data breaches. The largest number of incidents are reported in the medical and healthcare sector. The largest incident was reported by Yahoo on the 14th of December 2016, which compromised users’ data from three billion accounts. Table 2 shows the same statistics for inter-arrival times. We observe that the mean values of inter-arrival times are less than the standard deviations for each category. It provides evidence that inter-arrival times cannot be modelled with the Poisson distribution. A similar conclusion can be drawn for the breach sizes.

**Table 1:** Summary statistics of breach sizes

Type of organisation	Min	Median ($\times 10^3$)	Mean ($\times 10^6$)	Sd ($\times 10^6$)	Max ($\times 10^6$)	Number of observations
BSF	6	1.7	4.8	21.3	145.5	111
BSO	2	10.4	26.4	214.8	3000.0	208
BSR	1	2.1	6.7	33.3	327.0	138
EDU	12	8.5	222.5	2.7	40.0	223
GOV	8	6.0	457.7	2.4	21.5	93
MED	1	4.0	200.1	2.9	78.8	805
NGO	13	4.0	142.1	0.6	3.0	24
Total	1	4.6	4.5	78.6	3000	1602

**Table 2:** Summary statistics of breach inter-arrival times

Type of organisation	Min	Median	Mean	Sd	Max	Number of observations
BSF	0.0111	2.00	4.16	5.78	36	111
BSO	0.0480	1.00	3.08	4.18	38	208
BSR	0.0233	2.00	3.52	5.09	33	138
EDU	0.0134	3.00	5.86	8.12	59	223
GOV	0.0842	2.00	3.66	5.06	28	93
MED	0.0019	1.00	2.85	4.10	37	805
NGO	0.0131	1.00	2.70	3.56	13	24
Total	0.0019	2.00	3.49	5.20	59	1602

**Fig. 2:**
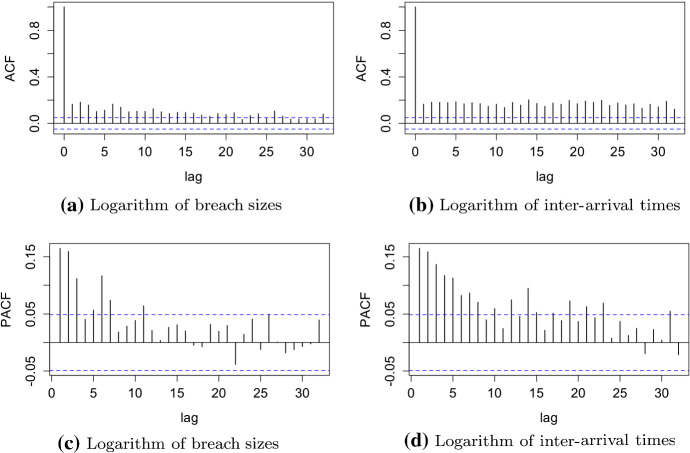
ACF and PACF

Analogously to Xu et al. (2018), we check auto-correlation (ACF) and partial auto-correlation functions (PACF) of the logarithm of breach size and logarithm of inter-arrival time. ACF measures the linear dependence between the lags of time-series, whereas PACF is the correlation between lags adjusted for the contributions of observations in between (Hyndman and Athanasopoulos 2018; Shumway and Stoffer 2016). These measures are used to find if observations exhibit a correlation between each other and can be modelled with a stochastic process. Figure 2 shows that both breach sizes and inter-arrival times exhibit significant auto-correlations above the threshold values depicted with dotted lines. It indicates that they can be modelled with stochastic processes.

#### Quantile autoregression

**Fig. 3:**
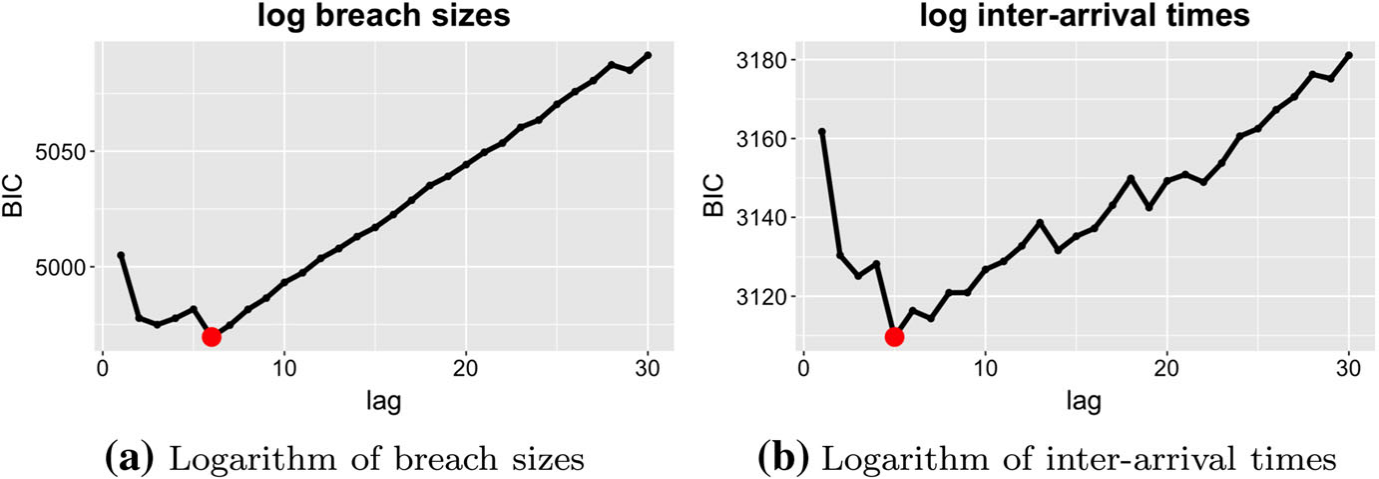
BIC for different lags

In this section, we model ${{\,\mathrm{VaR}\,}}_\alpha$ of the logarithm of breach sizes and the logarithm of inter-arrival times with QAR. First, we need to pick the optimal lag of QAR. Analogous to the problem of choosing the optimal degree of polynomial regression, the optimal order of the autoregressive process (2) can be chosen by some information criterion. We use the Bayesian Information Criterion (BIC) (Schwarz 1978) to pick the optimal lag of QAR. BIC is defined as follows:

$$\begin{aligned} \text {BIC} = -2\ln {L} + p \ln N, \end{aligned}$$

where *L* is the maximum of the model’s likelihood, *p* is the number of parameters, and *N* is the sample size. BIC penalises complex models with large lag number *p*, and smaller values of the criterion are favourable. Figure 3a shows BIC values for a different number of lags of QAR, which is built on the training data for quantiles equal to 0.5, i.e.  median values. The smallest values of BIC correspond to the optimal choice of the lag and are depicted with the red dots. We observe that the optimal values of lag are equal to six in the case of the breach size, and the optimal lag for the inter-arrival time is five.

**Fig. 4:**
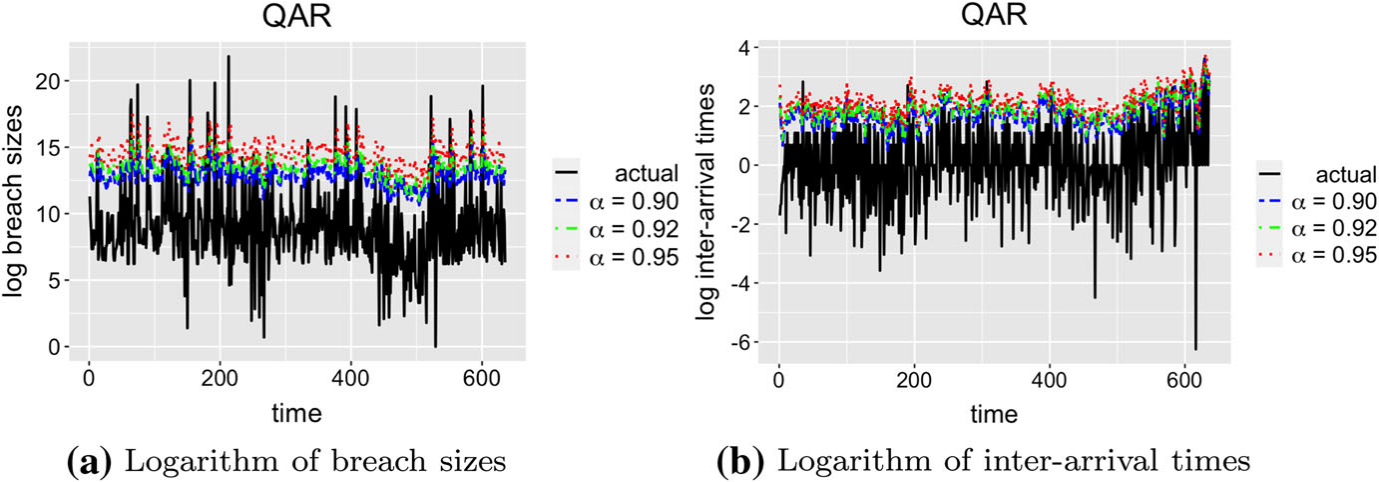
Predictions of QAR

We then build QAR for the optimal lags on the training dataset. These models are used for making predictions of ${{\,\mathrm{VaR}\,}}_\alpha$ on the test dataset. We pick the significance levels to be $\alpha = 0.9, 0.92, 0.95$. From the risk perspective, it is important to estimate how large the potential losses might be in order to prevent or hedge these losses. Therefore, $\alpha$ values should be large. Figure 4a, b illustrate the predictions of QAR for breach sizes and inter-arrival times respectively, on the test data.

If the observed value exceeds the predicted ${{\,\mathrm{VaR}\,}}_\alpha$, we call it *violation*. The Kupiec unconditional coverage test (Kupiec 1995) measures whether the number of violations is consistent with the confidence level. For example, if $\alpha = 0.9$, then the percent of observation, which exceeds the predicted ${{\,\mathrm{VaR}\,}}_{0.9}$, should be close to 0.1. The null hypothesis $H_0$ is that the observed violation rate is equal to $1 - \alpha$. The Kupiec unconditional coverage test focuses only on the number of violations. However, we would like to test whether these exceptions are evenly spread over time. The null hypothesis $H_0$ for the Christoffersen conditional coverage test (Christoffersen 1998) is that the probability of observing a violation at some time point does not depend on whether a violation occurred. Table 3 illustrates the results of backtesting of both coverage tests for breach size and inter-arrival time respectively, on the test data. The table shows the expected number of violations of the considered confidence level and the actual number of violations of the considered method. We use the following notations: exp (expected number of violations), act (actual number of violations), the unconditional coverage test p-value (uc.LRp), the conditional coverage test p-value (cc.LRp), the unconditional coverage test decision (uc.D), and the conditional coverage test decision (cc.D), fail to reject the null hypothesis $H_0$ (FR), reject the null hypothesis $H_0$ (R). We can see that QAR(6) fails to reject the null hypothesis $H_0$ for both unconditional and conditional coverage tests, which means that the models fit well and describe the quantiles of breach size correctly. Table 4 shows the case of inter-arrival time, QAR(5) fits well for 0.9 and 0.95 quantiles, however, for 0.92 the conditional coverage test rejects the null hypothesis. In the next section, we show how we can improve the prediction of the breach inter-arrival times by applying CQAR.

**Table 3:** Coverage tests for QAR for breach sizes at test data

Method	Quantile	Exp	Act	uc.LRp	cc.LRp	uc.D	cc.D
QAR(6)	0.90	63	55	0.2509	0.4784	FR	FR
QAR(6)	0.92	50	44	0.3095	0.5103	FR	FR
QAR(6)	0.95	31	29	0.6116	0.7456	FR	FR

**Table 4:** Coverage tests for QAR for inter-arrival times at test data

Method	Quantile	Exp	Act	uc.LRp	cc.LRp	uc.D	cc.D
QAR(5)	0.90	63	56	0.3062	0.3539	FR	FR
QAR(5)	0.92	50	41	0.1360	0.0146	FR	R
QAR(5)	0.95	31	26	0.2765	0.1463	FR	FR

#### Competitive quantile autoregression

In this section, we estimate the hacking breaches’ inter-arrival times with CQAR. In contrast to QAR, CQAR does not need a training dataset. The algorithm starts its training when it gets the first observation of the test dataset. However, as we have the training dataset available, we pick the regularisation parameter *a* and the standard deviation $\sigma$ from the training data. Table 5 illustrates the acceptance ratio and the total pinball loss of CQAR on the training dataset for different parameters *a* and $\sigma$. The lowest pinball loss on the training data is achieved with $a = 1$ and $\sigma = 0.7$, which is depicted in bold. The corresponding acceptance ratio for these parameters is 0.27. It is important to ‘track’ the acceptance ratio of CQAR. A very high acceptance ratio might indicate that the algorithm moves too slowly to the optimal parameter $\theta$. Therefore, the total number of iterations and the burn-in period should be increased. Another option is to increase the standard deviation $\sigma$. Table 5 shows that increasing $\sigma$ leads to decreasing of the acceptance ratio.

**Table 5:** Parameters of CQAR on training

(a) Acceptance ratio				(b) Pinball losses			
a \$$ \sigma $$	0.5	0.7	1	a \$$ \sigma $$	0.5	0.7	1
0.1	0.69	0.47	0.22	0.1	281.69	281.74	268.00
0.5	0.61	0.36	0.12	0.5	177.52	171.76	172.50
1	0.53	0.27	0.06	1	137.20	**135.42**	138.21

Table 6 shows the results of the backtesting for CQAR(5) on the test dataset. Note that even though we pick the parameters of the CQAR using the prior knowledge, the algorithm starts with zero parameters $\theta$ and trains using only the test dataset. We can see from the table that both unconditional and conditional coverage tests for CQAR(5) fail to reject the null hypothesis. Therefore, CQAR(5) produces better results for predicting breach inter-arrival times than QAR(5). The p-values of CQAR are also higher than p-values of QAR, apart from the cc.LRp for 0.90 quantile. The results are also on par with Table 8 of VaR tests of predicted inter-arrival times in Xu et al. (2018), though since then a significant amount of hacking breaches has been reported.

**Table 6:** Coverage tests for CQAR for inter-arrival times at test data

Method	Quantile	Exp	Act	uc.LRp	cc.LRp	uc.D	cc.D
CQAR(5)	0.90	63	69	0.4808	0.0785	FR	FR
CQAR(5)	0.92	50	54	0.6514	0.1025	FR	FR
CQAR(5)	0.95	31	27	0.3705	0.4844	FR	FR

**Fig. 5:**
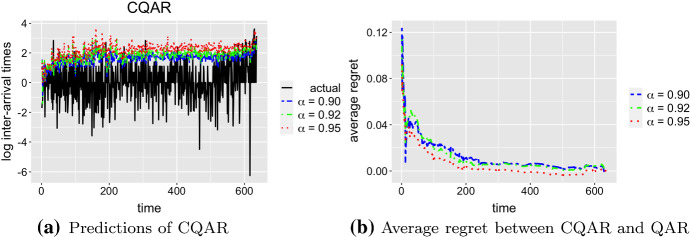
CQAR

The important property of CQAR is that it asymptotically predicts as well as any QAR. Figure 5a illustrates the predictions of CQAR for $\alpha = 0.9, 0.92, 0.95$ on the test data. Figure 5b shows the average regret between CQAR(5) and QAR(5). As we discussed, CQAR starts with zero parameters $\theta$ at the beginning of its training, and as a result, the average regret is high at the start. However, it becomes close to zero for all considered quantiles as time increases. The resulting graph confirms the theoretical behaviour of the average regret described in Lemma 1.

### Other types of data breaches

In this section, we focus on two other types of data breaches, each of which contributes to around a fifth of reported breaches: PHYS (information lost or stolen from paper documents) and DISC (unintended disclosure of sensitive information posted publicly, mishandled or sent to the wrong party). The three main objectives of these experiments are:

1. testing of CQAR performance on additional datasets;
2. comparing CQAR with other popular methods for time-series modelling, such as ARMA-GARCH, which was also used in Xu et al. (2018);
3. providing a more realistic scenario for a dynamic method, i.e. CQAR initially uses non-optimal parameters *a* and $\sigma$, and then these parameters are dynamically tuned on the past test data (training dataset is not used for the CQAR’s parameter tuning in this setting).

#### Physical loss of paper documents

In this subsection, we model the logarithm of data breaches sizes caused by a physical loss of paper documents. The total number of observations after removing all incomplete, unknown, and missing breaches is 1473. Interestingly, the majority of the reported cases are in the medical sector (89 %). Similar to previous experiments, we hold the first 60% of observations for the training of ARMA-GARCH. The rest of the data is used for training of CQAR and comparison of results. Similar to Xu et al. (2018), the mean of breach sizes is modelled with ARMA, whereas the volatility is estimated with GARCH.

Analogous to the previous experiment, we first examine ACF and PACF that exhibit a significant correlation between observations. Therefore, we can model the breach sizes with a stochastic process. In these experiments, we try to model the breach sizes with ARMA-GARCH that models both the mean and the volatility with different stochastic processes. First, we try to identify the optimal order of ARMA(p, q)-GARCH(m, n). Considering even three different values of four parameters is tiresome: it leads to 81 different combinations. Therefore, we consider that GARCH(1, 1) is enough to describe the volatility of breach sizes. GARCH(1, 1) is one of the most commonly used models for volatility modelling and it produces results that are often on par with the models of higher-order (Hansen and Lunde 2005). Table 7 shows BIC of different orders of ARMA(p, q), where the minimum is achieved at $p=q=1$ (shown in bold).

**Table 7:** BIC of different orders of ARMA(p, q)

p \ q	0	1	2
0	3608.2	3604.9	3605.1
1	3603.1	**3581.2**	3586.7
2	3602.1	3586.7	3593.0

**Fig. 6:**
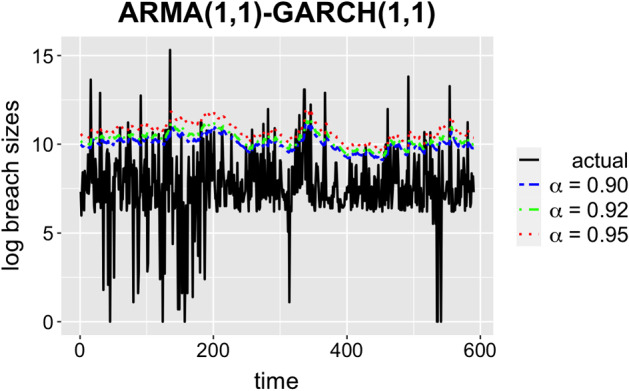
Predictions of ARMA(1, 1)-GARCH(1, 1)

As we found the optimal order of the model, we can fit ARMA(1, 1)-GARCH(1, 1) that produces a 1-step ahead prediction and re-trains every 50 steps. Figure 6 shows the predictions and Table 8 illustrates the coverage tests of the method. We can see that ARMA(1, 1)-GARCH(1, 1) provides a good fit for predicting the breach sizes. We now want to see how CQAR performs on the same test data.

**Table 8:** Coverage tests for ARMA(1, 1)-GARCH(1, 1) for breach sizes at test data

Method	Quantile	Exp	Act	uc.LRp	cc.LRp	uc.D	cc.D
ARMA-GARCH	0.90	58	51	0.2737	0.2510	FR	FR
ARMA-GARCH	0.92	47	40	0.2730	0.5399	FR	FR
ARMA-GARCH	0.95	29	25	0.3933	0.4830	FR	FR

Using BIC on the training dataset, we found that the optimal lag of QAR is equal to two. Now we use test data for CQAR training. Here we also consider a more realistic scenario for a dynamic method when we do not have any insight into the optimal parameters of CQAR. Therefore, we start with some random, non-optimal parameters *a* and $\sigma$, and then these parameters are dynamically tuned on a part of test data. In these experiments, we use the first quarter of the test data to estimate the optimal parameters of CQAR. We start with $a=\sigma =1$, after 25% of the outcomes is revealed, we estimate the optimal parameters to be $a= 0.5$ and $\sigma =0.7$ with the pinball loss 58.66 and the acceptance rate 41%. Table 9 shows the coverage tests for CQAR(2). We can see that CQAR(2) provides a worse fit in comparison to ARMA(1, 1)-GARCH(1, 1) as p-values are lower. In addition, for 90% quantile, it rejects the null hypothesis of both conditional and unconditional coverage tests. The use of non-optimal parameters might affect the performance of CQAR and might lead to a slower convergence.

**Table 9:** Coverage tests for CQAR(2) for breach sizes at test data

Method	Quantile	Exp	Act	uc.LRp	cc.LRp	uc.D	cc.D
CQAR(2)	0.90	58	41	0.0101	0.0361	R	R
CQAR(2)	0.92	47	35	0.0561	0.0674	FR	FR
CQAR(2)	0.95	29	22	0.1435	0.3333	FR	FR

#### Unintended disclosure of sensitive information

In this subsection, we repeat the same experiments for the prediction of inter-arrival times of data breaches caused by unintended disclosure of sensitive information posted publicly, mishandled or sent to the wrong party. The total number of observations after removing all incomplete, unknown, and missing breaches is 1552 with the first 60% of observations held for the training of ARMA-GARCH. Table 10 shows BIC of different orders of ARMA(p, q). As before, the minimum is achieved for $p=q=1$ (shown in bold), and therefore we use ARMA(1, 1)-GARCH(1, 1) for modelling. As in the previous experiment, we start to train CQAR(2) on the test data with non-optimal parameters $a=\sigma =1$ and after we see the first 25% of observations we re-estimate the parameters. The optimal parameters found are $a=0.7$ and $\sigma =1$ with the pinball loss 19.65 and the acceptance rate 35%. Table 11 illustrates the coverage tests of both methods. We can see from the table, that for quantiles 0.9 and 0.92, most of the p-values of ARMA(1, 1)-GARCH(1, 1) are higher than the ones of CQAR(2). However, CQAR(2) fails to reject all tests, whereas ARMA(1, 1)-GARCH(1, 1) rejects the null hypothesis for quantile 0.95.

**Table 10:** BIC of different orders of ARMA(p, q)

p \ q	0	1	2
0	3004.5	3002.2	2985.4
1	2999.0	**2920.8**	2926.9
2	2978.0	2926.8	2931.4

**Table 11:** Coverage tests of ARMA(1, 1)-GARCH(1, 1) and CQAR(2) for inter-arrival times at test data

method	quantile	exp	act	uc.LRp	cc.LRp	uc.D	cc.D
ARMA-GARCH	0.90	61	65	0.6801	0.4293	FR	FR
ARMA-GARCH	0.92	49	45	0.4969	0.7878	FR	FR
ARMA-GARCH	0.95	30	18	0.0097	0.0207	R	R
CQAR(2)	0.90	61	70	0.2867	0.3782	FR	FR
CQAR(2)	0.92	49	54	0.5125	0.0954	FR	FR
CQAR(2)	0.95	30	31	0.9926	0.0519	FR	FR

### Discussion

We provide an extensive experimental evaluation of proposed cyber risk estimation methods, QAR and CQAR. We show that both approaches provide a good fit to cyber breaches’ sizes and inter-arrival times. The comparison of CQAR with ARMA(1, 1)-GARCH(1, 1) illustrates that the methods are on par with each other even though CQAR uses a much smaller dataset for its training. ARMA(1, 1)-GARCH(1, 1) rejects the null hypothesis of both unconditional and conditional coverage tests for one quantile of data breaches caused by unintended disclosure of sensitive information. It is possible that if we tested the higher orders of GARCH it might have provided a better fit for the data. Another limitation is the choice of CQAR’s parameters. If we have some information on the optimal parameters the method converges faster and provides good results. However, when no information is available and we start with non-optimal parameters the method might not sample well. Therefore, it is important to keep track of the acceptance rate of the sampling parameters and re-estimate them when more data becomes available.

## Conclusions

In this paper, we have presented two approaches to cyber VaR estimation of time-series. VaR gives a prediction of extreme values with the desired confidence level for a different kind of time-series. These estimates can sequentially be translated into the monetary VaR, which is essential for budget planning and allocation. The first approach to estimate VaR is based on QAR, which provides a new way to model extreme values with the desired confidence level. QAR is more flexible compared to the previously proposed approaches as it allows to model VaR for each confidence level with a separate stochastic process, and hence relies on fewer assumptions on the nature of the data.

The second proposed approach, called CQAR, provides a new framework for dynamic cyber risk estimation. The method re-estimates VaR at each step as soon as new data becomes available. A significant property of this approach is the theoretical guarantee that it asymptotically performs as well as the best QAR found retrospectively. This important property provides confidence in the prediction as it will hold for any new unseen data, while at the same time the method allows adapting to a changing environment.

Finally, we demonstrate that both methods provide a good fit for predicting the size and inter-arrival times of different types of cyber breaches by running coverage tests. We show that CQAR asymptotically performs as well as the best QAR which conforms to the theoretical bounds of the method. The performance of CQAR also in par with ARMA(1, 1)-GARCH(1, 1). In addition, we provide a fully-reproducible code of our experiments.

## Appendix

### Lemma 2

(Lemma 2 in Levina et al. (2010)) Let $\lambda (y, \gamma ) \le L$ for all $y \in \varOmega$ and $\gamma \in \varGamma$. The Weak Aggregating Algorithm guarantees that, for all *T*

$$\begin{aligned} L_T \le \sqrt{T} \left( -\ln \int _{\varTheta } \exp \left( -\frac{L_T^{\theta }}{\sqrt{T}} \right) P_0 (d\theta ) + L^2 \right) . \end{aligned}$$

### Proof of Lemma 1

The proof is the same as the Proof of Theorem 1 from Dzhamtyrova and Kalnishkan (2020) adjusted for the time-series data. We provide the proof here for completeness.

Recall, that the learner makes a prediction $\gamma _t$ based on the signal $x_t = (1, y_{t-1}, \ldots , y_{t-p}) \in \mathbb {R}^{p+1}$, and outcomes come from the interval [*A*, *B*]. We choose an initial distribution of parameters (14):

$$\begin{aligned} P_0(d\theta ) = \left( \frac{a}{2} \right) ^{p+1} e^{-a \Vert \theta \Vert _1}d\theta , \end{aligned}$$

for some $a>0$, and $\theta \in \mathbb {R}^{p+1}$. Let us define the truncated prediction strategy $\tilde{\mathcal {E}}_{\theta }$ which at step *t* outputs:

18 $$\begin{aligned} \tilde{\xi }_t(\theta ) = {\left\{ \begin{array}{ll} A, &{} \text {if } x_t^\prime \theta < A \\ x_t^\prime \theta , &{} \text {if } A \le x_t^\prime \theta \le B \\ B, &{} \text {if }x_t^\prime \theta > B \end{array}\right. }. \end{aligned}$$

Let us denote $\tilde{L}^{\theta }_T$ the cumulative loss of prediction strategy $\tilde{\mathcal {E}}_{\theta }$ at the step *T*:

19 $$\begin{aligned} \tilde{L}^{\theta }_T := \sum _{t=1}^T \lambda (y_t, \tilde{\xi }_t (\theta )). \end{aligned}$$

We apply the algorithm’s prediction (16) with truncated prediction strategies $\tilde{\mathcal {E}}_{\theta }$. As prediction strategies output predictions inside the interval [*A*, *B*], and the algorithm’s prediction is a weighted average of strategies’ predictions, then $\gamma _t$ lies in the interval [*A*, *B*], $\forall t$.

We can bound the maximum loss at each time step:

20 $$\begin{aligned} L:= \max _{y \in [A,B],~\gamma \in [A, B]} \lambda (y, \gamma ) \le (B-A) \max (\alpha , 1-\alpha ) \le B-A. \end{aligned}$$

Lemma 2 provides the theoretical guarantees for the strategy that follows WAA (16) used by our algorithm. Applying Lemma 2 for initial distribution (14) and putting the maximum loss (20) we obtain:

21 $$\begin{aligned} L_T \le \sqrt{T} \left( -\ln \left( \left( \frac{a}{2} \right) ^{p+1} \int _{\mathbb {R}^{p+1}} e^{- \tilde{J}(\theta )} d\theta \right) + (B-A)^2 \right) , \end{aligned}$$

where

22 $$\begin{aligned} \tilde{J}(\theta ) := \frac{\tilde{L}_T^{\theta }}{\sqrt{T}} + a \Vert \theta \Vert _1. \end{aligned}$$

For all $\theta , \theta _0 \in \mathbb {R}^{p+1}$ we have:

23 $$\begin{aligned} \sum _{\begin{array}{c} t = 1, \ldots , T:\\ y_t< x_t^\prime \theta \end{array}} |x_t^\prime \theta - y_t|\le & {} \sum _{\begin{array}{c} t = 1, \ldots , T:\\ y_t< x_t^\prime \theta \end{array}} |x_t^\prime \theta _0 - y_t| + \sum _{\begin{array}{c} t = 1, \ldots , T:\\ y_t< x_t^\prime \theta \end{array}} |x_t^\prime \theta - x_t^\prime \theta _0|\nonumber \\\le & {} \sum _{\begin{array}{c} t = 1, \ldots , T:\\ y_t< x_t^\prime \theta \end{array}} |x_t^\prime \theta _0 - y_t| + \sum _{\begin{array}{c} t = 1, \ldots , T:\\ y_t< x_t^\prime \theta \end{array}} \smash {\displaystyle \max _{t=1,\ldots ,T}} \Vert x_t\Vert _{\infty } \Vert \theta - \theta _0\Vert _1 \nonumber \\\le & {} \sum _{\begin{array}{c} t = 1, \ldots , T:\\ y_t < x_t^\prime \theta \end{array}} |x_t^\prime \theta _0 - y_t| + T \max (1, B) \Vert \theta - \theta _0\Vert _1. \end{aligned}$$

Analogously, we have:

24 $$\begin{aligned} \sum _{\begin{array}{c} t = 1, \ldots , T:\\ y_t> x_t^\prime \theta \end{array}} |x_t^\prime \theta - y_t|\le & {} \sum _{\begin{array}{c} t = 1, \ldots , T:\\ y_t > x_t^\prime \theta \end{array}} |x_t^\prime \theta _0 - y_t| \nonumber \\&+ T \max (1, B) \Vert \theta - \theta _0\Vert _1. \end{aligned}$$

By multiplying inequality (23) by $(1-\alpha )$, inequality (24) by $\alpha$ and summing them, we have:

25 $$\begin{aligned} L_T^{\theta } \le L_T^{\theta _0} + T \max (1, B) \Vert \theta - \theta _0\Vert _1. \end{aligned}$$

The cumulative loss of truncated prediction strategy $\tilde{\mathcal {E}}_{\theta }$ cannot exceed the cumulative loss of non-truncated prediction strategy $\mathcal {E}_{\theta }$ for all $\theta \in \mathbb {R}^{p+1}$:

26 $$\begin{aligned} \tilde{L}_T^{\theta } \le L_T^{\theta } . \end{aligned}$$

By dividing (25) by $\sqrt{T}$ and adding $a \Vert \theta \Vert _1$ to both parts, we have:

27 $$\begin{aligned} \tilde{J}_T(\theta ) \le J_T(\theta )\le & {} J_T(\theta _0) + \sqrt{T} \max (1, B) \Vert \theta - \theta _0\Vert _1 + a \left( \Vert \theta \Vert _1 - \Vert \theta _0\Vert _1 \right) \nonumber \\\le & {} J_T(\theta _0) + \left( \sqrt{T} \max (1, B) + a\right) \Vert \theta - \theta _0\Vert _1, \end{aligned}$$

where

28 $$\begin{aligned} J_T(\theta ) := \frac{L_T^{\theta }}{\sqrt{T}} + a \Vert \theta \Vert _1. \end{aligned}$$

Let us denote $b_T =\sqrt{T} \max (1, B) + a$. We evaluate the integral:

By putting this expression in (21) we obtain:

$$\begin{aligned} L_T \le L_T^{\theta } +\sqrt{T} a \Vert \theta \Vert _1 + \sqrt{T} \Bigg ((p+1) \ln \left( 1 + \frac{\sqrt{T}}{a} \max (1, B) \right) + (B-A)^2 \Bigg ). \end{aligned}$$

By putting this expression in formula for the average regret (10) we obtain the theoretical bound from Lemma 1. $\square$

## Footnotes

1 https://privacyrights.org/data-breaches.

2 https://github.com/alan-turing-institute/dynamic_cyber_risk.
